# Calcium/calmodulin dependent protein kinase IV in trophoblast cells under insulin resistance: functional and metabolomic analyses

**DOI:** 10.1186/s10020-023-00669-8

**Published:** 2023-06-29

**Authors:** Ling Li, Li Li, Ying Shao, Runyu Du, Ling Li, Xiaoguang Shi, Yu Bai

**Affiliations:** Department of Endocrinology, Shengjing Hospital of China Medical University, 36 Sanhao Street, Shenyang, 110004 Liaoning People’s Republic of China

**Keywords:** Calcium/calmodulin dependent protein kinase IV, Gestational diabetes mellitus, Insulin resistance, Trophoblast cells, Autophagy, metabolomics

## Abstract

**Background:**

Insulin resistance (IR) is an important determinant of glucose metabolic disturbance and placental dysplasia in gestational diabetes mellitus (GDM). Calcium/calmodulin dependent protein kinase IV (CAMK4) improves insulin IR induced by a high-fat diet (HFD). The current study sought to elucidate the role and potential mechanism of CAMK4 in GDM.

**Methods:**

A GDM model was established in female C57BL/6J mice via HFD feeding for one week before mating and throughout gestation. The IR was elicited by 10^–6^ M insulin treatment for 48 h in HTR-8/SVneo cells and mouse primary trophoblast cells. The function of CAMK4 was investigated by transfection of overexpression plasmid in HTR-8/SVneo cells and infection of lentivirus loaded with CAMK4 encoding sequence in primary trophoblast cells. Real-time PCR, western blot, cell counting kit-8, transwell, wound healing, dual-luciferase reporter assay, and liquid chromatography/mass spectrometry-based untargeted metabolomics were performed to confirm the effects of CAMK4 on trophoblast cells.

**Results:**

Decreased CAMK4 expression was found in the placenta of GDM mice. CAMK4 overexpression ameliorated IR-induced viability impairment, migratory and invasive capacity inhibition, autophagy blocking, insulin signaling inactivation and glucose uptake disorder in trophoblast cells. CAMK4 also transcriptionally activated orphan nuclear receptor NUR77, and the effects of CAMK4 were abrogated by silencing of NUR77. Metabolomics analysis revealed that CAMK4 overexpression caused alterations of amino acid, lipid and carbohydrate metabolism, which were important in GDM.

**Conclusion:**

Our results indicated that CAMK4/NUR77 axis may provide novel potential targets in GDM treatment.

**Supplementary Information:**

The online version contains supplementary material available at 10.1186/s10020-023-00669-8.

## Introduction

Gestational diabetes mellitus (GDM) is a frequent pregnancy complication, in which women without previously diagnosed diabetes develop hyperglycemia in the second or third trimester of pregnancy (American Diabetes A 2021). This hyperglycemia is the consequence of abnormal glucose tolerance since β-cell insulin secretion is unable to compensate for the progressive insulin resistance (IR) during pregnancy (Buchanan and Kitzmiller 1994). GDM increases the risk for future maternal and offspring cardiovascular disease and type 2 diabetes. Pre-pregnancy overweight/obesity, advanced age, and a family history of IR and/or diabetesare common risk factors for GDM (Plows et al. 2018). The prevalence of GDM continues to rise worldwide given the escalating epidemic of obesity and diabetes (Sweeting et al. 2022). Although some management for GDM exists, including lifestyle modifications (diet and exercise) and insulin therapy, there is not yet an efficient cure or prevention strategy for GDM due to the presence of IR.

IR is an important determinant of glucose metabolic disturbance and placental dysplasia in hyperglycemia/GDM (Catalano et al. 2003). Placental trophoblasts are vital to the development of the placenta during pregnancy. Inhibition of trophoblast cell viability, invasion, and migration may contribute to the maldevelopment of placental tissues, resulting in recurrent spontaneous abortion (Zong et al. 2016; Tian et al. 2015). Increasing insulin sensitivity facilitates trophoblast cell migration (Mayama et al. 2013).

Calcium/calmodulin dependent protein kinase IV (CAMK4), which belongs to the serine/threonine kinase family, and to the Ca^2+^/calmodulin-dependent protein kinase subfamily. CAMK4 is a multifunction protein, acts as a transcription regulator or a kinase (Liu et al. 2020; Bhargava et al. 2021). *Camk4*^*−/−*^ mice exhibited hypertension, mitigated inflammation, ion dyshomeostasis and neuron dysfunction (Santulli et al. 2012; Yong et al. 2022; Sabbir 2020; Ribar et al. 2000; Wei et al. 2002). CAMK4 was also demonstrated to participate in whole-body improvements of insulin sensitivity (Lee et al. 2014). CAMK4 enhanced insulin-stimulated muscle glucose uptake with an increase in insulin-sensitive glucose transporter GLUT4 expression and activation of insulin signaling. Moreover, CAMK4 contributed to hepatic and adipose insulin responsiveness via an increase in myokines released from the skeletal muscle (Lee et al. 2014). CAMK4 also improved impaired insulin sensitivity in high-fat diet (HFD) mice through the induction of hepatic autophagy (Liu et al. 2020). Li and colleagues found that lowly expressed CAMK4 inhibited proliferative and migratory abilities in HTR-8/SVneo trophoblast cells (Li et al. 2020). At present, the effect of CAMK4 on GDM remains unknown. It deserves our attention whether CAMK4 ameliorates GDM via increasing insulin sensitivity.

Orphan nuclear receptor NUR77, also known as NR4A1, involves in various biological processes, including trophoblast cell differentiation, invasion, and embryonic development (Malhotra and Gupta 2017; Shen et al. 2020; Li et al. 2019a). NUR77 has been reported to induce gene expression associated with glucose metabolism in skeletal muscle (Chao et al. 2007). Deletion of NUR77 also exacerbates HFD-elicited IR in both skeletal muscle and liver of mice, accompanied by impaired insulin signaling and reduced GLUT4 protein expression (Chao et al. 2009). We previously found that overexpression of NUR77 relieved IR in HTR-8/SVneo cells via activation of autophagy and insulin signaling (Li et al. 2022). In addition, highly expressed NUR77 promoted the proliferation, migration and invasion of HTR-8/SVneo cells under IR condition (Li et al. 2022). Blaeser et al. revealed that CAMK4 induced NUR77 transcription by directly binding to the MEF2 response elements on the NUR77 promoter (Blaeser et al. 2000). However, whether CAMK4 controls IR in trophoblast cells via regulating NUR77 expression is undetermined.

Metabolites are the end products of transcription, translation and proteome and therefore reflect pathophysiologic functions from cells to tissues (Juppner et al. 2017). Metabolomics analysis is widely used to elucidate the pathogenesis of disease by identifying metabolic profiles (Johnson et al. 2016). Recent studies have examined metabolite alterations in the trophoblast cells and revealed some potential biomarkers via nontargeted metabolomics analysis (Kawasaki et al. 2019; Chen et al. 2022), which provides more direct information on the pathogenesis of the dysregulated placenta. In the present study, we assessed CAMK4 expression in the placenta tissues of HFD mice during pregnancy and the trophoblast cells under IR condition. By a series of functional experiments, the underlying mechanism of CAMK4 was further explored. Finally, we sought to characterize CAMK4-regulated metabolomics profiles in the HTR-8/SVneo cells under IR condition through liquid chromatography/mass spectrometry (LC–MS)-based untargeted metabolomics.

## Materials and methods

### Animal model

**Fig. 1:**
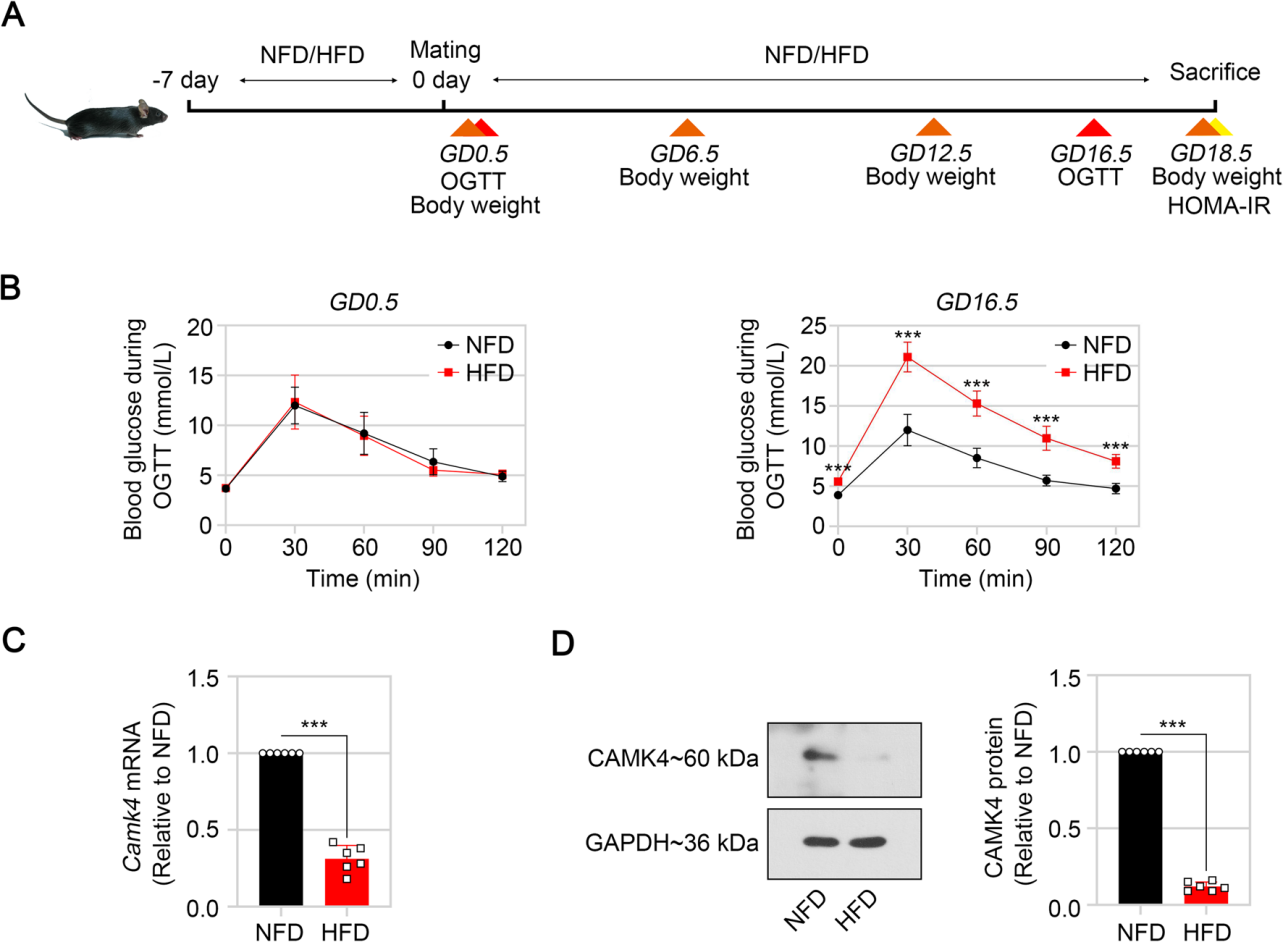
Low CAMK4 expression was found in the placenta of HFD mice during pregnancy. **A** An illustration of the experiments was shown. **B** Results of oral glucose tolerance test (OGTT) of these mice at GD0.5 and GD16.5 after fasting overnight (12 h). **C** and **D** The mRNA and protein levels of CAMK4 in the placenta of HFD mice at GD18.5 were detected by real-time PCR (**C**) and western blot (**D**), respectively. Error bars depict the standard deviation of the mean. NFD, normal-fat diet; HFD, high-fat diet; ***p < 0.001

Male and female C57BL/6 J mice at 8 weeks of age were housed in standard conditions (12-h light/12-h dark cycle, 23 ± 1 °C of temperature, 45–55% of humidity) with free access to food and water. After adaptation for one week, mice were fed a HFD (45% kcal from fat; Cat no., XTHF45, XieTongPharmaceutical Bio-Engineering, Nanjing, China) or normal-fat diet (NFD) (23% kcal from fat; Cat no., XTCON50H, XieTongPharmaceutical Bio-Engineering) for one week according to previous report (Liu et al. 2019). Female and male mice were bred overnight in a 2:1 ratio, and mating was evidenced by the presence of a vaginal mucous plug in females on the next morning, which was regarded as gestation day (GD)0.5. Pregnant mice were continuously fed HFD or NFD until GD18.5. An oral glucose tolerance test (OGTT) was conducted on GD0.5 and 16.5. The body weight of the pregnant mice was monitored on GD0.5, 6.5, 12.5 and 18.5. After fasting overnight (12 h), the blood were collected at GD18.5 from pregnant mice through cardiac puncture after anesthesia with 3% isoflurane inhalation. After blood collection, the mice died naturally. The placenta tissues was isolated for subsequent detections. An illustration of the experiments was shown in Fig. 1A. All experimental procedures were performed in accordance with national institutes of health guidelines for the care and use of animals and were approved by the institutional animal care and use committee at animal research center of Shengjing Hospital of China Medical University (Approval ID 2022PS384K).

### OGTT

Blood glucose of the pregnant mice was measured after fasting overnight (12 h) using a glucometer (Sinocare, Changsha, Hunan, China). Afterward, the mice were orally gavaged with 2 g/kg body weight of D-(+)-glucose (Cat no., G6152, Sigma-Aldrich, St. Louis, MO, USA), and the blood glucose was detected 30, 60, 90 and 120 min after intragastric administration.

### Homeostasis model assessment for insulin resistance (HOMA-IR)

Serum samples were collected after centrifugation at 1000 g for 20 min, and insulin levels was detected using a Mouse Insulin ELISA Kit (Cat no., EM0260, Fine Biotech., Wuhan, China), following the manufacturer’s protocol. The fasting blood glucose level was determined with a glucometer at GD18.5 before blood collection. The HOMA-IR was then calculated according to formula: HOMA-IR = (fasting insulin in mIU/L × fasting glucose in mmol/L)/22.5

### Cell culture and treatment

Human trophoblast cell line HTR-8/SVneo was obtained from Procell, Wuhan, China. The cells were cultured in RPMI-1640 media supplemented with 5% fetal bovine serum and maintained at 37 °C in a humidified 5% CO_2_ atmosphere. To assess whether CAMK4 was related to IR in GDM, HTR-8/SVneo cells were treated with 10^–6^ mol/L recombinant human insulin (Baiying Biotech., Shenyang, China) for 48 h to induce IR (Li et al. 2022). Subsequently, 10^–7^ mol/L insulin for 15 min was used to stimulate the insulin signaling.

HTR-8/SVneo cells at 70% confluence were transfected for 24 h with 2.5 μg plasmid overexpressing CAMK4 (oeCAMK4, Sino Biological, Beijing, China) or empty vector control using lipofectamine 3000 (Invitrogen, Carlsbad, CA, USA), according to the manufacturer’s instructions. For rescue experiments, a total of 1.25 μg plasmid was transfected alone or co-transfected with 37.5 pmol small interference RNAs targeting human NUR77 (siNUR77-1, siNUR77-2) using lipofectamine 3000. A non-targeting siRNA was used as a negative control (siNC). Insulin treatment was performed 24 h after transfection.

To observe the autophagic flux, an adenovirus vector carrying LC3 encoding sequence, green fluorescent protein and red fluorescent protein (Ad-mRFP-GFP-LC3) was delivered in HTR-8/SVneo cells (2 × 10^9^ PFU/mL). The merged yellow spots indicated autophagosomes, and red spots indicated autolysosomes.

To block the autophagic flux, chloroquine (CQ, Macklin, Shanghai, China) was applied in HTR-8/SVneo cells with 20 μM.

The primary trophoblast cells were isolated from mouse placental tissues, and cultured with special primary epithelial cell medium in a humid incubator with 37 °C and 5% CO_2_. The trophoblast cells were pre-treated with 10^–6^ mol/L insulin for 48 h to induce IR, and followed with a stimulation of 10^−7^ mol/L insulin for 15 min to active insulin signaling.

To realize the overexpression of CAMK4 and knockdown of NUR77, the lentivirus vector carring *mus Camk4* encoding sequence or silencing fragment targeting *mus Nur77* were used to infect the mouse primary trophoblast cells (1 × 10^8^ TU/mL).

### Immunofluorescent staining

The mouse primary trophoblast cells were identified by immunofluorescent staining of cytokeratin 7 (CK7). The cells were cultured on glass slides, and fixed with 4% (m/v) paraformaldehyde (Sinopharm, Shanghai, China) for 15 min. After rinsing with PBS, the cells were permeated with 0.1% (m/v) TritonX-100 (Beyotime, Shanghai, China) for 30 min, rinsed with PBS, blocked with 1% bovine serum albumin (Sangon, Shanghai, China) for 15 min, and incubated with antibody against CK7 (1:50, Cat no., A4357, ABclonal, Wuhan, China) at 4 °C overnight. Subsequently, the cells were incubated with goat anti-rabbit IgG labeled with Cy3 (1:200, Cat no., A27039, Invitrogen, Carlsbad, CA, USA) at room temperature for 60 min in the dark, followed with counterstaining of DAPI. Finally, the glass slides were sealed with anti-fading reagent (Solarbio, Beijing, China), and images were taken with a fluorescent microsope at magnification 400 ×.

### Cell counting kit-8 (CCK-8) assay

The CCK8 assay was carried out to evaluate cell viablity. The cells (3 × 10^3^) in 100 μL medium per well were seeded into the 96-well plate. A total of 10 μL CCK-8 solution (KeyGene Biotech., Nanjing, China) was added to each well and incubated at 37 °C under 5% CO_2_ for 48 h. Finally, the OD value at 450 nm was measured using a microplate reader (BioTek, Winooski, VT, USA).

### Wound healing assay

Cell migrating ability was assessed by wound healing assay. The cells were maintained in the serum-free medium and treated with 1 μg/mL mitomycin C for 1 h to reach confluence. The monolayer was scratched using a 200 μL pipette tip and subsequently, the cell debris was rinsed with the serum-free medium. Images at 0 h and 24 h were captured with a phase-contrast microscope at magnification 100 ×. The migration rate was calculated as the width difference of the wound at 0 hand 24 h divided by the wound width at 0 h.

### Transwell assay

Cell invasion assay was performed using a Transwell system equipped with 8-µm-pore polycarbonate filters. The cells were re-suspended in the serum-free medium and loaded into the upper chamber of the filter, which was pre-coated with Matrigel (Corning, Inc., Corning, NY, USA). The filters were then placed into the 24-well plate containing the basal medium containing 10% FBS and incubated for 24 hat 37 °C under 5% CO_2_ in a humidified incubator. Cells that invaded beyond the lower surface of the filters were fixed with 4% paraformaldehyde, stained with 0.4% crystal violet dye, and observed under an inverted microscope at magnification 200 ×. The invading number of cells was counted in five randomly chosen microscopic fields.

### Dual-luciferase reporter assay

HTR-8/SVneo cells seeded in 12-well plates were co-transfected with 1 μg pGL3-Basic reporter carrying NUR77 promoter fragment (− 1800 ~ + 2), 1 μg pRL-TK, and 1 μg oeCAMK4 or vector using lipofectamine 3000, according to the manufacturer’s instructions. Luciferase activity was assayed 24 h after transfection with a dual luciferase reporter assay kit (Cat no., KGAF040, KeyGene Biotech) and normalized to the*Renilla* luciferase activity.

### Real-time PCR

Total RNA was extracted from the placental tissues or cells using the TRIpure reagent. The BeyoRT II M-MLV Reverse Transcriptase was used for cDNA synthesis. Real-time PCR was performed using the cDNA template, SYBR Green and primers. GAPDH was regarded as the endogenous internal control. The relative expression level of each target gene was calculated using the 2^−ΔΔCT^ method. The sequences of primers were shown as follows:*homo CAMK4* (forward) 5′-GCGATCAGTTCATGTTCAG-3′,

(reverse) 5′-GTAGTCAGCCGTTTCTTTG-3′;*mus Camk4* (forward) 5′-ATCCACCTTCAACCCAA-3′,

(reverse) 5′-GATCCTGAGGCACCATAC-3′;*homo NUR77* (forward) 5′-GCAAGTGGGCGGAGAAGA-3′,

(reverse) 5′-CCAGGCGGAGGATGAAGAG-3′;*mus Nur77* (forward) 5′-CCTGGCATACCGATCTAAAC-3′,

(reverse) 5′-AGGCGGGAACATCAACAC-3′;*homo GAPDH* (forward) 5′-GACCTGACCTGCCGTCTAG-3′,

(reverse) 5′-AGGAGTGGGTGTCGCTGT-3′;*mus Gapdh* (forward) 5′-TGTTCCTACCCCCAATGTGTCCGTC-3′,

(reverse) 5′-CTGGTCCTCAGTGTAGCCCAAGATG-3′.

### Glucose consumption detection

Glucose level in the cell supernatant was measured using the Glucose Assay Kit (Cat no., F006, Jiancheng Bioengineering Institute) according to the manufacturer’s protocol. A BCA protein assay kit (Cat no., P0011; Beyotime) was used for the detection of protein concentration.

### Western blot

Whole proteins were extracted from the placental tissues or cells using the RIPA lysis. The protein concentration was determined using a BCA protein assay kit according to the manufacturer’s protocol (Cat no., P0011; Beyotime). Equal amounts (20–40 μg) of lysate proteins were separated by sodium dodecyl sulfate–polyacrylamide gel electrophoresis and transfected onto PVDF membranes. After blocking with 5% (m/v) skimmed milk for 1 h at room temperature, the membranes were incubated with primary antibodies at 4 °C overnight, followed by secondary antibodies at 37 °C for 45 min. Antibody information was as follows: anti-CAMK4 (Cat no., A5304, ABclonal; 1:2000), anti-NUR77 (Cat no., A6676, ABclonal; 1:1000), anti-Beclin 1 (Cat no., A7353, ABclonal; 1:500), anti-p62 (Cat no., A19700, ABclonal;1:1000), anti-LC3 (Cat no., A5618, ABclonal; 1:1000), anti-insulin receptor substrate-1 (IRS-1) (Cat no., AF6273, Affinity, Cincinnati, OH, USA; 1:500), anti-p-IRS1 Ser307 (Cat no., AF3272, Affinity; 1:500), anti-p-IRS1 Tyr612 (Cat no., AP0553, ABclonal; 1:500), anti-AKT (Cat no., AF6261, Affinity; 1:1,000), anti-p-AKT Ser473 (Cat no., AF0016, Affinity; 1:1000), anti-GAPDH (Cat no., 60004-1-Ig, ProteinTech, Rosemont, IL, USA; 1:10,000), horseradish peroxidase conjugated goat anti-rabbit IgG (Cat no., A0208, Beyotime; 1:5000) and anti-mouse IgG (Cat no., A0216, Beyotime; 1:5000). The blots were visualized using enhanced chemiluminescence and quantified by Gel-Pro-Analyzer software.

### Untargeted metabolomics analysis of polar metabolites

To examine the CAMK4-induced metabolomic alterations in trophoblasts upon IR stimulation, the HTR-8/SVneo cells were transfected with oeCAMK4 or vector for 24 h, and then treated with 10^–6^ mol/L recombinant human insulin for 48 h (n = 6 in each group). All LC–MS/MS experiments and the metabolome analyses were performed by PANOMIX Biomedical Tech Co., LTD (Suzhou, China). The cells (1 × 10^7^ cells) were treated with pre-cooled acetonitrile: methanol: H_2_O mixed solution (2:2:1, v/v/v) and the resulting extract was analyzed by LC–MS/MS (positive and negative modes) using liquid chromatograph (Vanquish, ThermoFisher Scientific, Pittsburgh, PA, USA) and mass spectrometer (Orbitrap Exploris 120, ThermoFisher Scientific). Peak extraction and alignment were performed by R XCMS program. The metabolites were identified by the self-built database and the public databases Human Metabolome Database (HMDB) (http://www.hmdb.ca), massbank (http://www.massbank.jp/), LipidMaps (http://www.lipidmaps.org), mzcloud (https://www.mzcloud.org) and Kyoto Encyclopedia of Genes and Genomes (KEGG) (http://www.genome.jp/kegg/). The quality control-based robust locally estimated scatterplot smoothing signal correction (QC-RLSC) was used for data normalization to correct for any systematic bias. Only ion peaks with relative standard deviations (RSDs) below 30% in QC were kept to ensure proper metabolite identification. The identified metabolites were subjected to further statistical analysis to screen for metabolites affected by CAMK4 overexpression in HTR-8/SVneo cells upon IR stimuli. Orthogonal projections to latent structures discriminant analysis (OPLS-DA) was carried out to evaluate the metabolic alterations and selectpotential metabolites [variable importance on projection (VIP) value > 1]. Differentially expressed metabolites (DEMs) were subsequently selected with the following criteria: VIP value > 1 and p < 0.05.

### Differential metabolite pathway analysis

MetaboAnalyst was used to analyze KEGG pathway enrichment of CAMK4-regulated metabolites. The metabolites and corresponding pathways were visualized using KEGG Mapper tool.

### Statistical analysis

Data were analyzed using GraphPad Prism 6.0 software by analysis of variance (ANOVA) followed by Tukey’s for multiple comparisons; unpaired two‑tailed Student’s t‑test for two groups. p < 0.05 was consideredstatisticallysignificant. All results are expressed as mean ± standard deviation (SD).

## Results

### Low CAMK4 expression was found in placenta of HFD mice during pregnancy

The gestational diabetes was induced in mice as shown in Fig. 1A. Maternal body weight was determined at GD0.5, 6.5, 12.5, and 18.5. Compared with the NFD mice, HFD mouse weight rapidly increased throughout the pregnancy (Additional file 1: Figure S1A). OGTT results exhibited a worse response to glucose in HFD mice at all time points (30, 60, 90, and 120 min) (Fig. 1B). During OGTT at GD16.5, fasting blood glucose levels of HFD mice were significantly higher than those of NFD mice (Fig. 1B). HFD mice developed IR as exhibited by the increased fasting glucose level, fasting insulin levels and HOMA-IR at GD18.5 (Additional file 1: Figure S1B).

At GD18.5, HFD mice displayed lower CAMK4 mRNA and protein levels in placental tissues than NFD mice (Fig. 1C).

### CAMK4 overexpression increased viability, accelerated migration and invasion of HTR-8/SVneo cells under IR condition

**Fig. 2:**
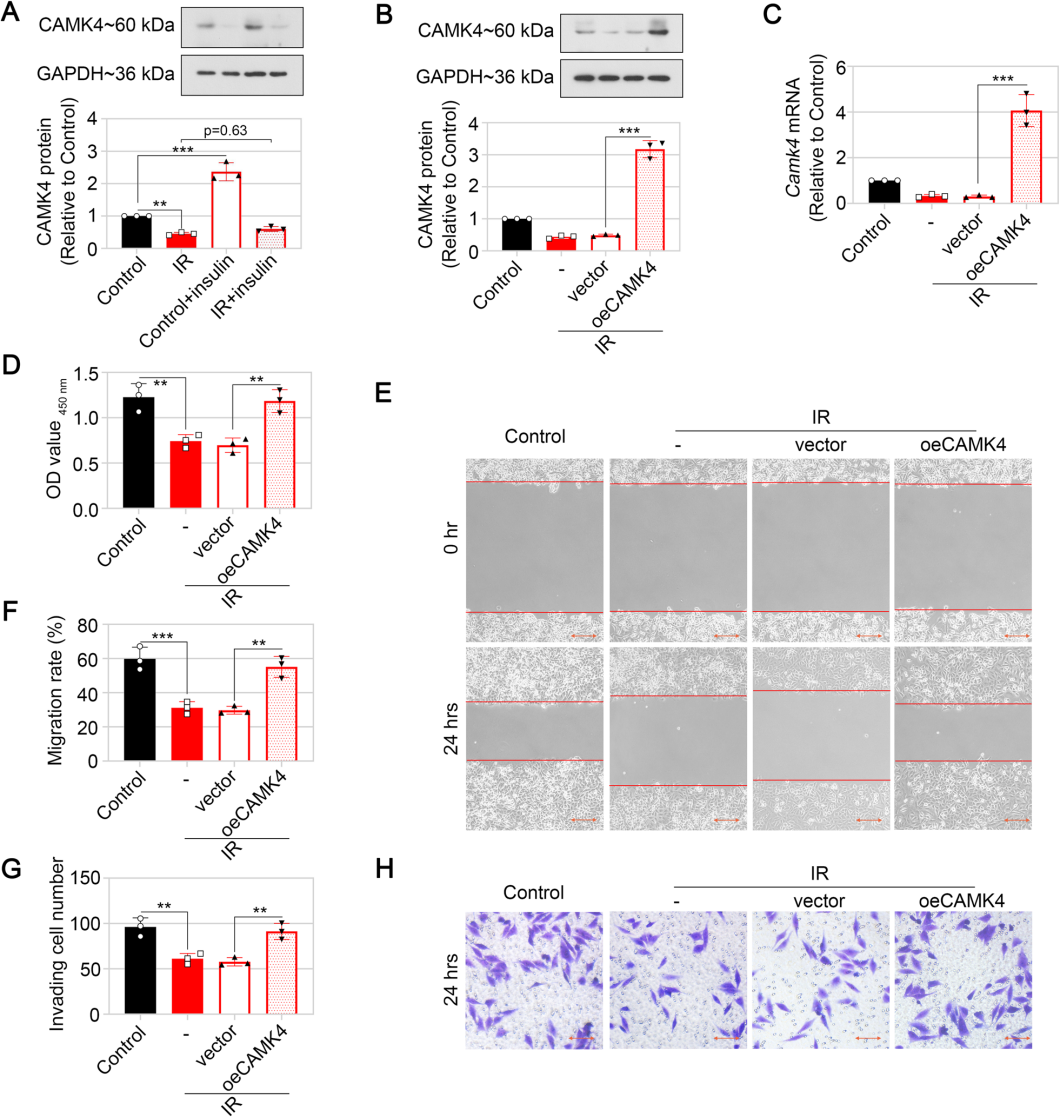
CAMK4 overexpression increased viability, accelerated migration and invasion of HTR-8/SVneo cells under IR condition. **A** HTR-8/SVneo cells were pre-incubated with or without 10^–6^ mol/L insulin for 48 h, followed by stimulation with 10^−7^ mol/L insulin for 15 min. The protein levels of CAMK4 in HTR-8/SVneo cells were detected by western blot. **B** and **C** Insulin pre-incubation was performed 24 h after transfection of CAMK4 overexpression plasmid or empty vector. The protein and mRNA levels of CAMK4 in HTR-8/SVneo cells were detected by western blot (**B**) and real-time PCR (**C**), respectively. **D** The viability of HTR-8/SVneo cells was determined by CCK-8 assay. **E** and **F** The migratory capacity of HTR-8/SVneo cells was determined by wound healing assay. Scale bar = 200 μm (**E**). The wound width was measured at 0 h and 24 h post-scratching, and the migration rate was calculated (**F**). **G** and **H** The invasive capacity of HTR-8/SVneo cells was determined by matrigel-based transwell assay. Scale bar = 100 μm (**H**).The number of cells that invaded beyond the lower surface of the membrane was counted (**G**). Error bars depict thestandard deviation of the mean. IR, insulin resistance; −, no treatment; ^ns^ no significance, **p < 0.01, ***p < 0.001

To explore the function of CAMK4 in GDM-related IR at cellular level, HTR-8/SVneo cells were pre-incubated with 10^–6^ mol/L insulin for 48 h to elicit IR condition. CAMK4 level was decreased in the IR cells compared with the control cells (Fig. 2A). Stimulation with 10^–7^ mol/L insulin for 15 min enhanced CAMK4 level in the control cells (Fig. 2A; Control vs. Control + insulin), but yielded no effect on CAMK4 level in the IR cells (Fig. 2A; IR vs. IR + insulin).

In order to confirm the roles of CAMK4, the overexpression plasmid was tranfected into HTR-8/SVneo cells, and the expression effectiveness was confirmed at both mRNA and protein levels (Fig. 2B and C). Results from CCK-8, wound healing and transwell assays revealed that IR led to suppression of proliferation, migration and invasion of HTR-8/SVneo cells, while these alterations were reversed by CAMK4 overexpression (Fig. 2D–H), similarly with previous report (Li et al. 2020).

### CAMK4 overexpression triggered autophagy in HTR-8/SVneo cells under IR condition

**Fig. 3:**
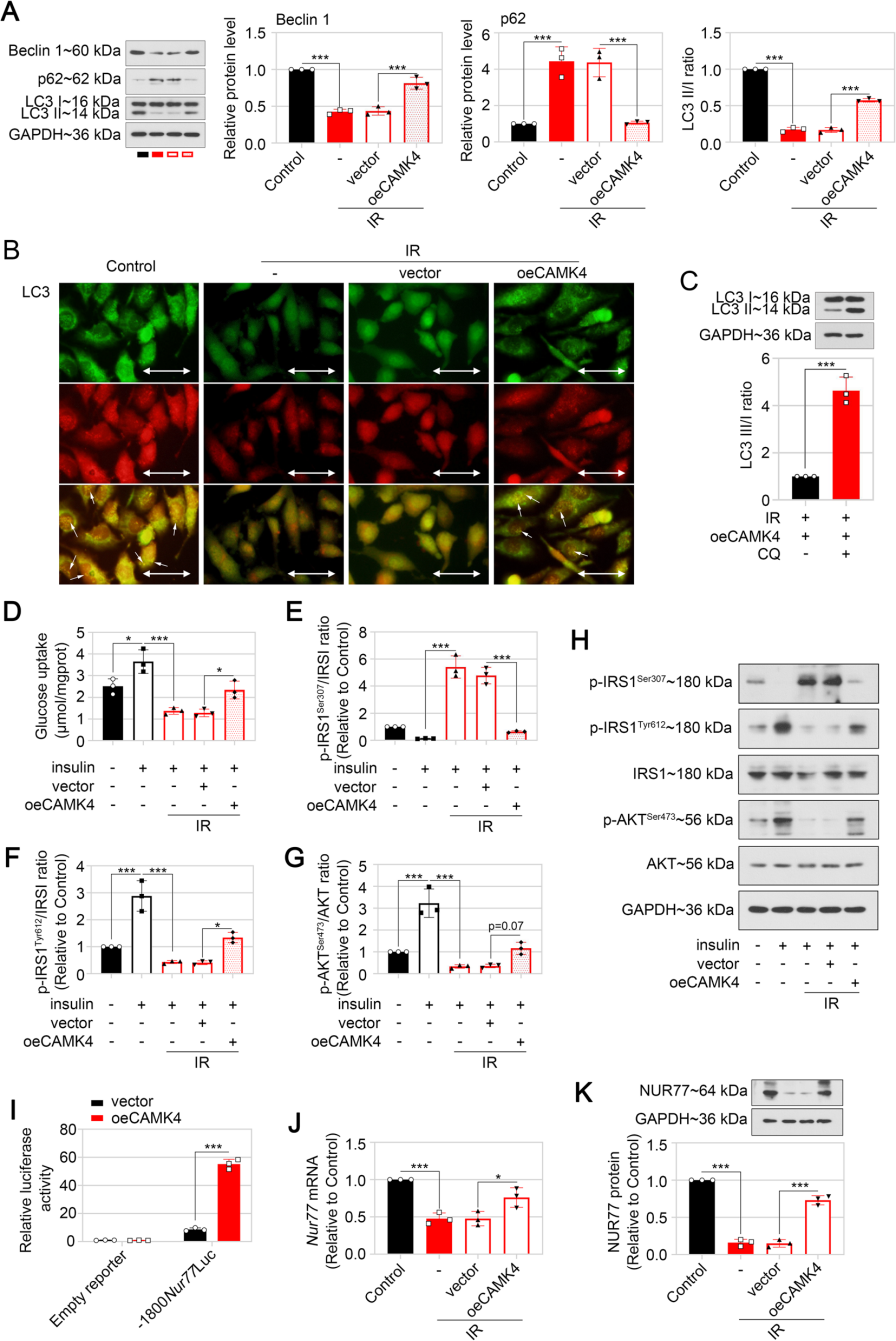
CAMK4 overexpression triggered autophagy and inhibited IR in HTR-8/SVneo cells under IR condition. HTR-8/SVneo cells were transfected with CAMK4 overexpression plasmid or vector for 24 h, followed by incubation with or without 10^–6^ mol/L insulin for 48 h (**A–C**). **A** Representative western blot images and relative protein levels of Beclin 1, p62, LC3 I and LC3 II in HTR-8/SVneo cells were displayed. **B** HTR-8/SVneo cells were infected with adenovirus vector carrying mRFP-GFP-LC3, and the red/green fluorescence was observed. Scale bar = 50 μm. **C** The levels of LC3 I/II in HTR-8/SVneo cells with 10^–6^ mol/L insulin pre-treatment, CAMK4 overexpression or/and CQ administration. HTR-8/SVneo cells were pre-incubated with or without 10^–6^ mol/L insulin for 48 h, followed by stimulation with 10^–7^ mol/L insulin for 15 min, and the transfection of CAMK4 overexpression plasmid or empty vector was performed 24 h before insulin pre-incubation (**D–H**). **D** Glucose uptake ability was assessed in cells of each group. **E–H** Theratio of Phospho.IRS-1^Ser307^/Total.IRS-1 (**E**), Phospho.IRS-1^Tyr612^/Total.IRS-1 (**F**) or Phospho.AKT^Ser473^/Total.AKT (**G**) in HTR-8/SVneo cells wasdetected by western blot. Representative western blot images of phosphorylated IRS-1 at Ser307, phosphorylated IRS-1 at Tyr612, total IRS-1, phosphorylated AKT at Ser473, total AKT were displayed (**H**). **I** Dual-luciferase reporter assay was performed to assess the effect of CAMK4 overexpression on luciferase activity of NUR77 in HTR-8/SVneo cells. **J** and **K** HTR-8/SVneo cells were pre-incubated with or without 10^−6^ mol/L insulin for 48 h, and the transfection of CAMK4 overexpression plasmid or empty vector was performed 24 h before insulin pre-incubation. The mRNA and protein levels of NUR77 in HTR-8/SVneo cells were detected by real-time PCR (**J**) and western blot (**K**), respectively. Error bars depict the standard deviation of the mean. IR, insulin resistance; −, no treatment; CQ, chloroquine; *p < 0.05, ***p < 0.001

Autophagy inhibition in trophoblast cells was previously demonstreated to induce pregnancy loss by blunting trophoblast invasion (Tan et al. 2020). Our data showed that the expression of autophagy-associated protein Beclin 1 was decreased, p62 was increased, and the LC3 II/I ration was also reduced in IR HTR-8/SVneo cells, suggesting impaired autophagy (Fig. 3A). While enhanced expression of CAMK4 promoted Beclin 1 expressio and conversion of LC3 I to LC II, and inhibited p62 levels (Fig. 3A). To observe the autophagic flux, an Ad-mRFP-GFP-LC3 vector was delivered into HTR-8/SVneo cells. When the autophagosome fused with the lysosome, the green fluorescent disappeared. As shown in Fig. 3B, the merged yellow spots indicated autophagosomes, and red spots indicated autolysosomes. Similarly with western blot results, the autophagy in HTR-8/SVneo cells was inhibited by IR conditions, and triggered by CAMK4 expression (Fig. 3B). In addition, when CQ was applied, the excessively accumulated LC3 II indicated blocked autophagic flux induced by CAMK4 in IR cells (Fig. 3C). These results suggested that CAMK4 overexpression activated autophagy in HTR-8/SVneo cells under IR condition.

### Upregulation of CAMK4 inhibited IR in HTR-8/SVneo cells

It has been reported that disruption of insulin signaling transmission causes defects in glucose uptake and results in IR (Zhou et al. 2022). Insulin signaling and glucose uptake ability were measured in the IR cellular model to determine whether CAMK4 was associated with IR in trophoblast cells. As shown in Fig. 5A, CAMK4 overexpression promoted insulin sensitivity by stimulating glucose uptake in the IR cells. Moreover, CAMK4 overexpression reduced insulin-stimulated phosphorylation of IRS-1 at Ser307 (inactivation site) and enhanced phosphorylation of IRS-1 at Tyr612 (activation site) and AKT at Ser473 in the IR cells (Fig. 3D–H), suggesting that upregulation of CAMK4 blunted insulin signaling disruption.

### CAMK4 transcriptionally activated NUR77 in HTR-8/SVneocells under IR condition

Our previous study revealed that NUR77 inhibited IR in HTR-8/SVneo cells through the activation of autophagy-mediated insulin signaling (Li et al. 2022). In addition, CAMK4 was known to promote NUR77 transcription (Blaeser et al. 2000). To elucidate whether NUR77 was required for CAMK4-caused IR inhibition in trophoblast cells, we detected the luciferase activity of NUR77 promoter sequence-loading vector after transfection of oeCAMK4 or vector via the dual-luciferase reporter assay. The results suggested that CAMK4 overexpression increased the luciferase activity of dual-luciferase reporter carrying NUR77 promoter sequence in HTR-8/SVneo cells (Fig. 3I). By performing real time-PCR and western blot analysis, we found that CAMK4 overexpression enhanced the expression of NUR77 at both transcription and translation levels in HTR-8/SVneo cells under IR condition (Fig. 3J and K).

### NUR77 was required for CAMK4-mediated activation of autophagy and insulin signaling in HTR-8/SVneo cells

**Fig. 4:**
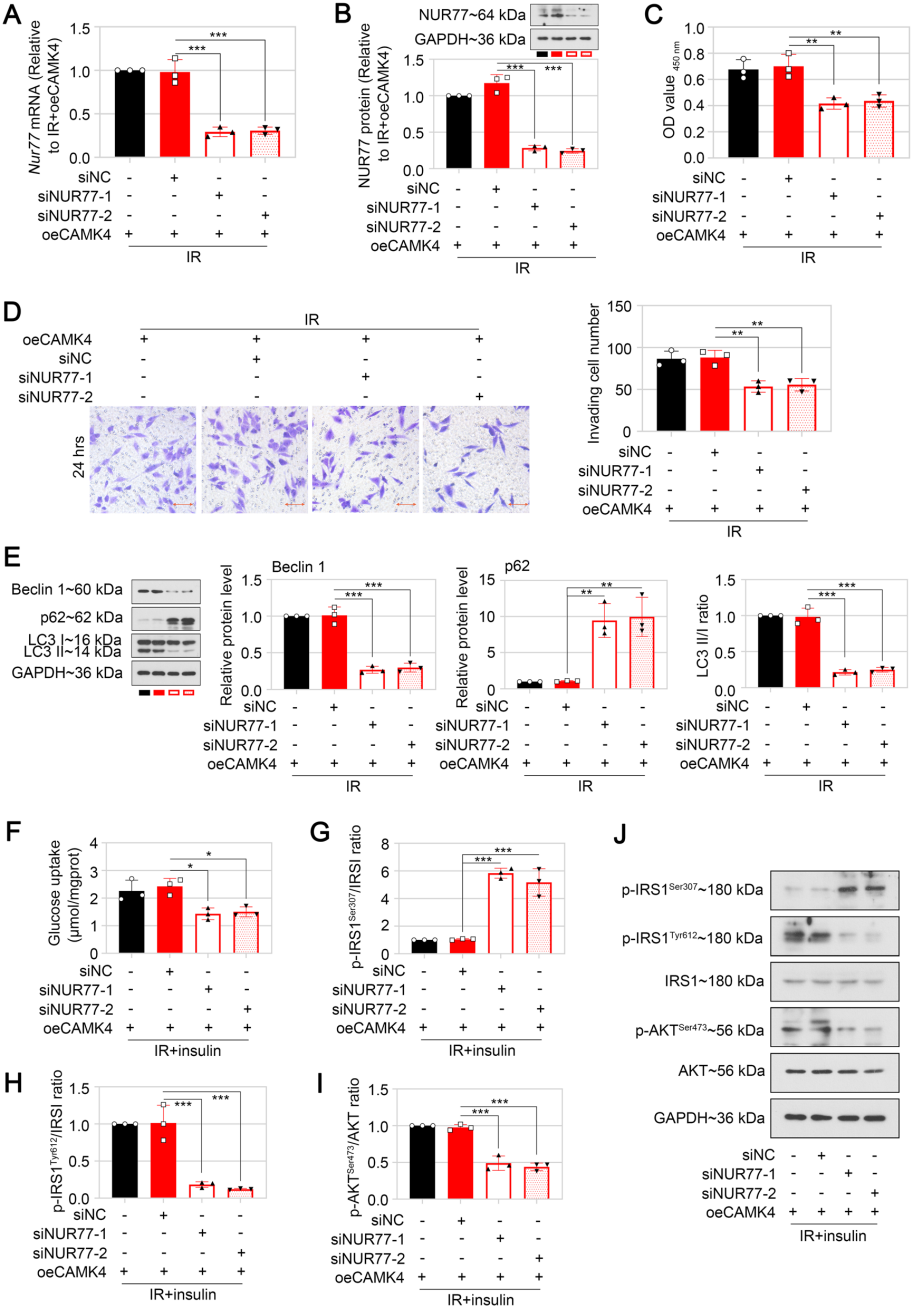
NUR77 was required for CAMK4-mediated activation of autophagy and insulin signaling in HTR-8/SVneo cells. HTR-8/SVneo cells were transfected with CAMK4 overexpression plasmid and siNUR77 or siNC for 24 h, followed by incubation with 10^–6^ mol/L insulin for 48 h. **A** and **B** The mRNA and protein levels of NUR77 in HTR-8/SVneo cells were detected by real-time PCR (**A**) and western blot (**B**), respectively. **C** The proliferation of HTR-8/SVneo cells was determined by CCK-8 assay. **D** The invasive capacity of HTR-8/SVneo cells was determined by matrigel-based transwell assay. Scale bar = 100 μm. **E** The expression of autophagy-associated proteins in HTR-8/SVneo cells was detected by western blot. **F** Glucose uptake ability was assessed in cells of each group. **G–J** The ratio of Phospho.IRS-1^Ser307^/Total.IRS-1 (**G**), Phospho.IRS-1^Tyr612^/Total.IRS-1 (**H**) or Phospho.AKT^Ser473^/Total.AKT (**I**) in HTR-8/SVneo cells was detected by western blot. Representative western blot images of phosphorylated IRS-1 at Ser307, phosphorylated IRS-1 at Tyr612, total IRS-1, phosphorylated AKT at Ser473, total AKT were displayed (**J**). Error bars depict the standard deviation of the mean. IR, insulin resistance; −, no treatment; *p < 0.05,**p < 0.01, ***p < 0.001

Next, the HTR-8/SVneo cells were transfected with CAMK4 overexpression plasmid and siNUR77 or siNC, followed by incubation with 10^–6^ mol/L insulin for 48 h. Real time-PCR and western blot analyses demonstrated that knockdown of NUR77 reduced NUR77 expression in IR cells transfected with oeCAMK4 (Fig. 4A and B). The biological behaviors in trophoblast cells were assessed by CCK-8 and transwell assays. Pro-proliferative and pro-invasive roles of CAMK4 overexpression in IR cells were inhibited by knocking down NUR77 (Fig. 4C and D).

To determine whether CAMK4 was a cause of NUR77-mediated IR in HTR-8/SVneo cells, the autophagy- and insulin signaling-associated indicators were detected by western blot. Knockdown of NUR77 reversed CAMK4 overexpression-induced autophagy activation, as evidenced by enhanced p62 expression and reduced Beclin 1 expression and LC3 II/I ratio (Fig. 4E). Furthermore, decreased glucose uptake and disrupted insulin signaling in IR cells were observed following co-transfection of oeCAMK4 and siNUR77 (Fig. 4F–J).

### Ectopic expression of CAMK4 promoted proliferation, invasion and autophagy in primary trophoblast cells by upregulating NUR77 expression

**Fig. 5:**
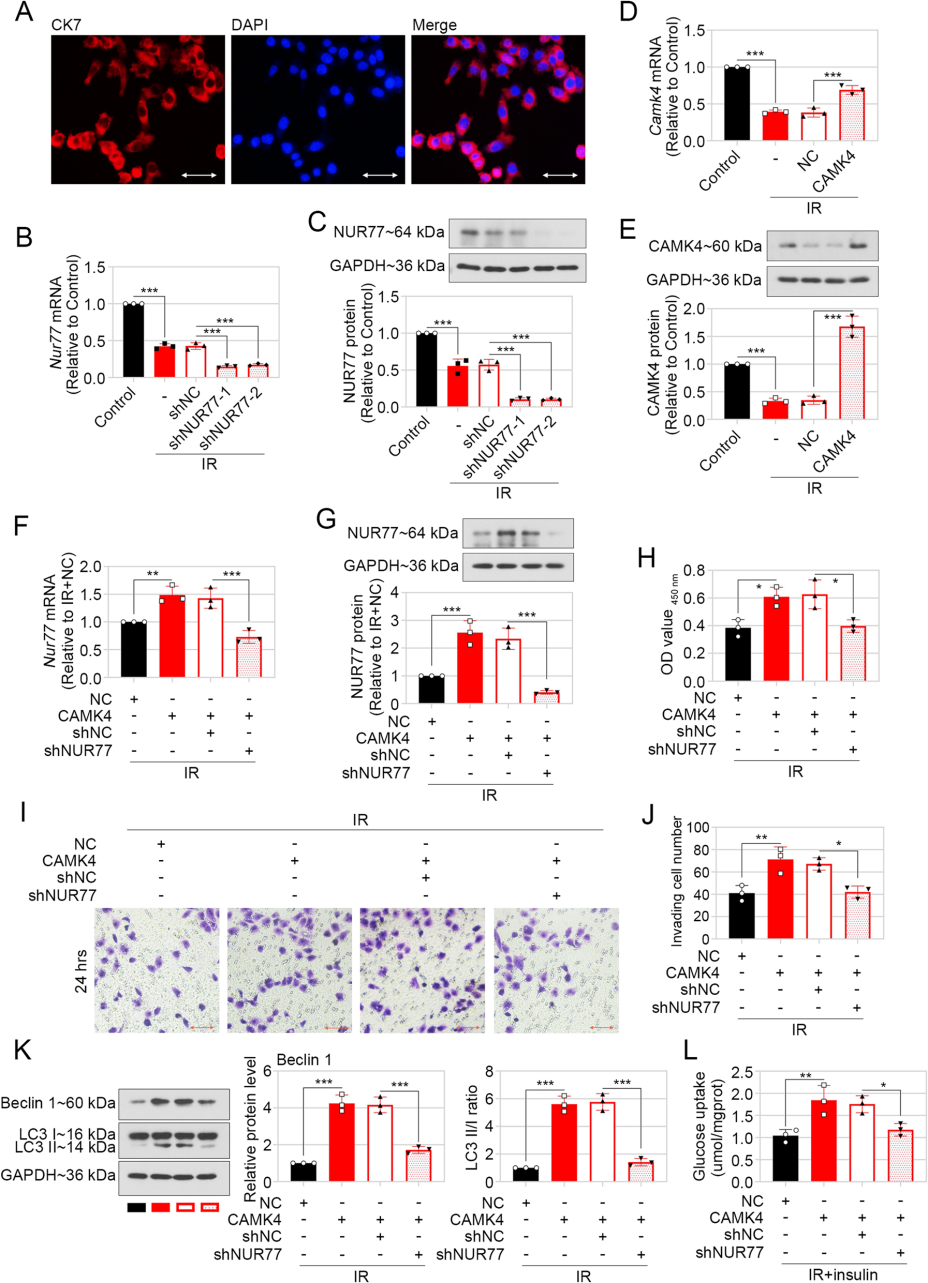
CAMK4 overexpression promoted invasion and autophage by upregulating NUR77 expression in primary trophoblast cells under IR conditions. **A** The mouse primary trophoblast cells were identified by staining of CK7. The trophoblast cells were infected with lentivirus vector carrying CAMK4 encoding sequence or/and knockdown sequence targeting *mus* NUR77, followed by incubation with or without 10^–6^ mol/L insulin for 48 h. **B** and **C** The mRNA and protein levels of NUR77 were determined in trophoblast cells with IR treatment and silencing of NUR77. **D** and **E** The mRNA and protein levels of CAMK4 in trophoblast cells with IR treatment and CAMK4 overexpression. **F** and **G** The mRNA and protein levels of NUR77 were assessed by real-time PCR (**F**) and western blot (**G**), respectively. **H** The proliferation of trophoblast cells was measured by CCK-8 assay. **I** and **J** The invasive capacity of trophoblast cells was examined by matrigel-based transwell assay. Scale bar = 100 μm. **K** Representative western blot images and relative protein levels of Beclin 1, LC3 I/II in trophoblast cells were exhibited. **L** Glucose uptake ability was assessed in trophoblast cells with CAMK4 overexpression, NUR77 knockdwon or/and IR induction and insulin stimulation. Error bars depict the standard deviation of the mean. IR, insulin resistance; −, no treatment; *p < 0.05,**p < 0.01, ***p < 0.001

To further confirm the roles of CAMK4 and NUR77 in trophoblast cells, the primary trophoblast cells were isolated from mouse placental tissues, and identified by staining of CK7 (Fig. 5A). The lentivirus vector loaded with CAMK4 encoding sequence or silencing fragment targeting NUR77 was delivered into trophoblast cells, and the effectiveness of lentivirus vector was confirmed by real-time PCR and western blot (Fig. 5B–E). The upregulation of CAMK4 on NUR77 was also confirmed in primary trophoblast cells under IR conditions (Fig. 5F, G), similarly with results in HTR-8/SVneo cells. The results from CCK-8 and transwell assays revealed that enhanced expression of CAMK4 accelerated proliferation and invasion, which were reduced by silencing of NUR77 in IR trophoblast cells (Fig. 5H–J). Immunoblotting results displayed that overexpressing CAMK4 caused increased Beclin 1 expression and LC3 II/I ratio, indicating promoted autophagy, which was reversed by NUR77 knockdown in IR trophoblast cells (Fig. 5K). The glucose uptaken of trophoblast cells was also facilitated by CAMK4 ectopic expression and reduced by NUR77 silencing under IR condition (Fig. 5L).

### CAMK4 overexpression induced metabolic alterations in HTR-8/SVneo cells under IR condition

**Fig. 6:**
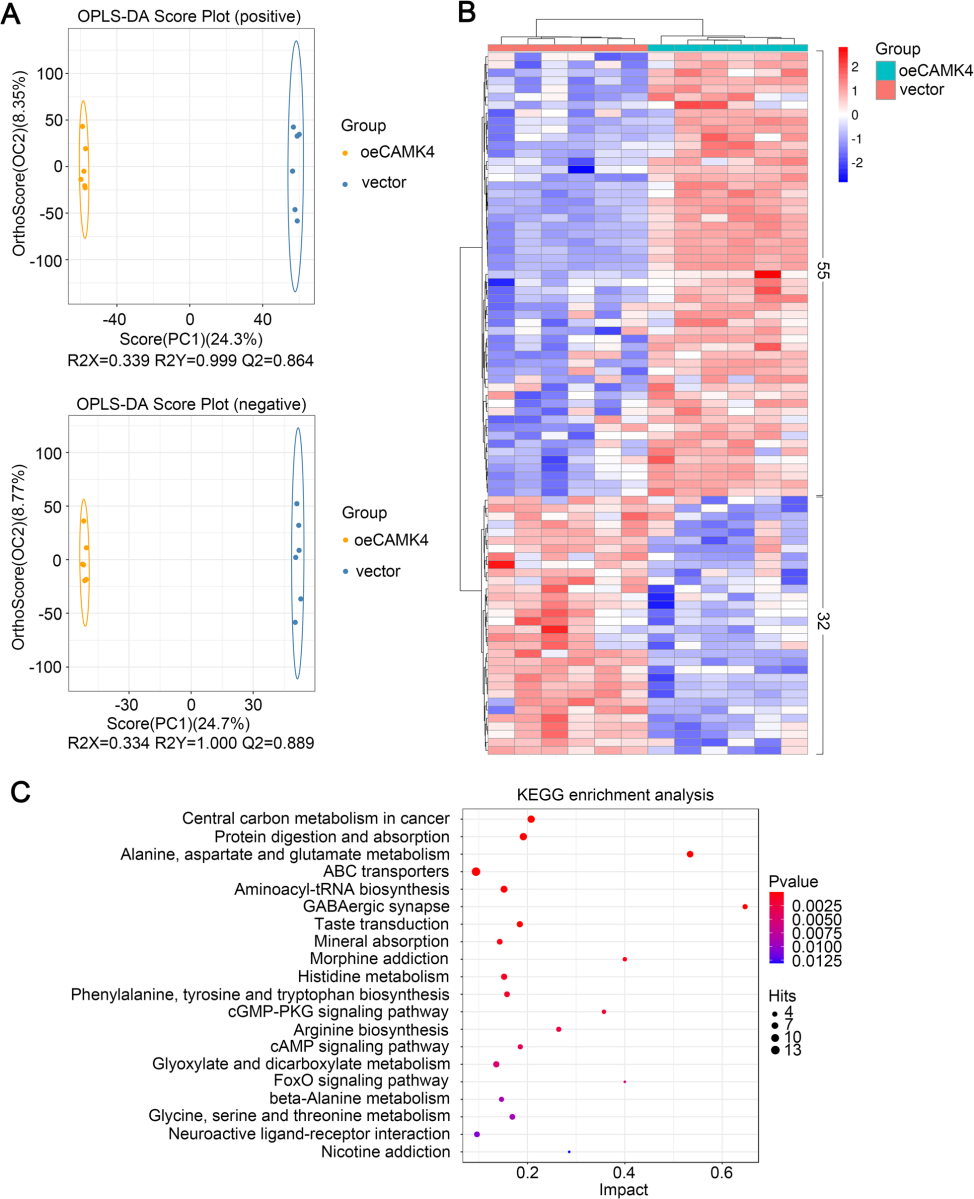
CAMK4 overexpression induced metabolic alterations in HTR-8/SVneo cells under IR condition. **A** Orthogonal projections to latent structures discriminant analysis (OPLS-DA) score plots in positive (upper) or negative (lower) ion mode. **B** Clustering heat map of 87 differentially expressed metabolites (DEMs), including 55 upregulated and 32 downregulated metabolites. **C** Kyoto Encyclopedia of Genes and Genomes (KEGG) pathway analysis result of all DEMs

We determined that CAMK4 overexpression improved autophagy and IR in HTR-8/SVneo cells. To assess the effect of CAMK4 overexpression on metabolites in HTR-8/SVneo cells under IR condition, the untargeted metabolomics analysis of polar metabolites was performed on IR-treated HTR-8/SVneo cells after transfection of oeCMAK4 or vector (six biological replicates for each group). The polar metabolites contains hydrosoluble amino acids, carbohydrates, lipid, nucleobases, vitamin and their derivatives. The OPLS-DA score plots demonstrated a clear distinction between vector samples and oeCAMK4 samples in the first principal component (Fig. 6A). A total of 4236/3267 unique metabolites were detected after CMAK4 overexpression, under positive/negative ion mode (Additional file 3: Table S1, Additional file 4: Table S2). Based on self-built database and the public databases, the LC–MS/MS analysis further identified 87 DEMs, including 55 up-regulated and 32 down-regulated metabolites (VIP value > 1 and p < 0.05) (Additional file 5: Table S3). The relative levels of DEMs in vector and oeCAMK4 samples were visualized on a heat map (Fig. 6B). KEGG pathway analysis showed that “Central carbon metabolism in cancer”, “Protein digestion and absorption”, “Alanine, aspartate and glutamate metabolism”, “ABC transporters”, and “Aminoacyl-tRNA biosynthesis” were the main KEGG pathways enriched by the DEMs (Fig. 6C). In the alanine, aspartate and glutamate metabolism pathway, l-glutamic acid and γ-aminobutyric acid (GABA) were key metabolites, which were highly expressed in oeCAMK4 cells under IR condition (Additional file 5: Table S3).

## Discussion

Understanding the mechanism of CAMK4 in the development of GDM is of both scientific and clinical significance. A previous study has suggested that abnormally expressed CAMK4 impaired trophoblast functions, but its role in trophoblasts has not been fully clarified (Li et al. 2020). In this study, we sought to characterize the role of CAMK4 on trophoblast cells under IR condition. Our data demonstrated that CAMK4 overexpression increased viability, migration and autophagy, and induced the insulin signaling activation of trophoblast cells. The improvement of insulin sensitivity in trophoblast cells may be associated with the restoration of NUR77-mediated autophagy.

An intracellular process sensitive to glucose levels is autophagy, a self-degradative mechanism that has been associated with the progression of metabolic diseases (Kim and Lee 2014). Autophagy plays a crucial role in trophoblast functions, including invasion and vascular remodeling in extravillous trophoblasts (EVTs), to promote normal placental development (Nakashima et al. 2013). Autophagy suppression induces the failure of trophoblastic invasion and results in poor placentation in trophoblast-specific autophagy related (Atg)7 knockout mouse model (Aoki et al. 2018). It has been revealed that autophagy inhibition is linked to the development of GDM (Nakashima et al. 2019). Analysis of the autophagy markers LC3, Beclin-1 and p62 shows impaired autophagy process in placentas from GDM pregnancies (Avagliano et al. 2017). Muralimanoharan et al. found that the deletion of autophagy gene Atg7 in placental tissue exhibited obvious placental abnormalities and sensitivity to HFD-induced obesity and hyperglycemia (Muralimanoharan et al. 2016). These findings indicate that activation of autophagy may contribute to the improvements of glucose metabolism and trophoblast function in GDM. In our previous report, HFD caused increase of LC3 II/I ratio, but reduction of Beclin 1 expression and accumulation of p62 in placental tissues of GDM mice, suggesting a defective autophagy, which was consistent with previous reports. The autophagy was also suppressed in trophoblast cells with IR. Therefore, we speculated that a moderate autophagy in placenta may be required during normal pregnancy. Moreover, the moderate autophagy may be elicited by physiological insulin level, and blocked or damaged by high insulin level.

An earlier study revealed that CAMK4 restored insulin sensitivity by triggering hepatic autophagy in obese mice (Liu et al. 2020). The silencing of CAMK4 curbed proliferative and migratory abilities in HTR-8/SVneo trophoblast cells (Li et al. 2020). In the current study, by performing the *in-vitro* functional experiments, we observed that CAMK4 overexpression relieved IR-induced autophagy blocking in tropholast cells, and then enhanced cell viability and migration. The restored autophage may contribute to the improvement of insulin sensitivity in CAMK4-overexpressed trophoblast cells.

Our previous report revealed that overexpression of NUR77 induces trophoblast cell invasion and enhances glucose uptake via triggering autophagy (Li et al. 2022). The results in the current study demonstrated that CAMK4 positive regulated the transcription of NUR77, and the effects of CAMK4 on trophoblast cells with IR were abolished by silencing of NUR77, suggesting that CAMK4 may promoted the invasion and autohpagy of trophoblast cells, an enhanced their insulin sensitivity through upregulating NUR77.

In addition, CAMK4 was reported to function by regulating other molecules. For instance, CAMK4 induced insulin gene expression by upregulating activating transcription factor-2 (ATF2) transcription activity in islet β ells (Ban et al. 2000). CAMK4 modulated podocyte motility by phosphorylating the scaffold protein 14-3-3β (Maeda et al. 2018). It is possible that CAMK4 regulate IR and autophagy via other pathway. However, it is so difficult to confirm the function of its every downstream effector. Our study revealed that CAMK4 function in trophoblast cells partically at least via regulating the transcription of NUR77. CAMK4 and NUR77 may be considered as potential targets in the treatment of GDM.

In our previous study, NUR77 was found highly expressed in placenta of HFD mice, but lowly expressed in HTR-8/SVneo cells under IR condition (Li et al. 2022). Moreover, NUR77 was demonstrated to be positively regulated by CAMK4 in cells, but their expression changes in placenta of GDM mice were not consistent. There seems to be some contradictions. Since overexpression of NUR77 promoted proliferation and invasion of IR trophoblast cells, we assumed that the upregulation of NUR77 in GDM mice was protective. As for why CAMK4 was decreased and NUR77 was increased in GDM mice, we hypothesized that NUR77 may be controlled by factors other than CAMK4 and CAMK4 was also regulated by several known or unknown molecules. Perhaps CAMK4 and NUR77 should be considered as two independent protective factors in GDM.

HTR-8/SVneo is an immortalized cell line that established from normal human first trimester placenta tissue (Graham et al. 1993), and used as trophoblast cells in a large number of studies. In our study, HTR-8/SVneo cells were induced IR to mimic the gestational diabetes environment in vivo. However, after multiple passages, the phenotypes of this cell line may undergo some uncertain changes. In order to better simulate the alterations in vivo, the mouse primary trophoblast cells were isolated, and also induced IR. Our results showed that high insulin concentration (10 nM) induced inactivation of insulin signaling and blocked glucose uptake, and low insulin concentration (1 nM) re-activated the insulin signaling. In normal mice, the blood insulin level was about 0.5 ng/mL, close to 1 nM. The blood insulin level of GDM mice was increased by about twofold, which was lower than insulin level of IR induction. We speculated that HFD-induced moderate increase of insulin level may be enough for IR elicitation in a long time (25 days) in mice. However, higher insulin concentration was essential for IR in cells in a short time (48 h). If the insulin concentration in mice is higher, it is believed that the IR would appear faster. IR may in turn induce compensatory elevation of insulin level, creating a vicious cycle. In addition, IR could also be triggered by high glucose concentration or combined treatment of high glucose and insulin in vivo in other reports (Du et al. 2022; Li et al. 2019b), suggesting that IR may be an inevitable phenotype of diabetes. Therefore, it remains further experiments to confirm whether the alterations of CAMK4/NUR77 expression and autophagy are associated with IR or hyperglycemia or hyperinsulinemia in the future.

It is essential for EVTs to invade into endometrium and myometrium during pregnancy, and an epithelial-to-mesenchymal transition (EMT) contributes to the invasive ability of trophoblast cells (Davies et al. 2016). The original HTR-8/SVneo cells were epithelioid, but after passages, they shifted a mesenchymal phenotype under normal culture conditions (Graham et al. 1993; Chen et al. 2013). It was reported that HTR-8/SVneo cell line contains a mixed populations of epithelial and mesenchymal cells (Abou-Kheir et al. 2017). In our study, the expression of several epithelial or mesenchymal markers were measured in HTR-8/SVneo cells after IR stimulation. As shown in Additional file 2: Figure S2, the E-cadherin and CK7 levels were increased and vimentin and TGF-β1 levels were decreased, suggesting a suppressed EMT in insulin resistant HTR-8/SVneo cells. It was reported that insulin induced EMT of mammary epithelial cells (Rodriguez-Monterrosas et al. 2018), while blocking insulin-like growth factor (IGF)/insulin-like growth factor-1 factor (IGF1R) signaling axis suppressed EMT of osteosarcoma cells (Cao et al. 2020). Our data firstly demonstrated that IR stimulation blocked EMT process of HTR-8/SVneo cells, may due to inactivation of insulin signaling, which may contributes to maldevelopment of placental tissues in GDM patients or animals.

We also found that CAMK4 overexpression induced the polar metabolomic profile alteration in HTR-8/SVneo cells under IR condition. Previous reports exhibited prominent alterations of amino acids, lipids and carbohydrates in amniotic fluid or serum of GDM women (Alesi et al. 2021; O'Neill et al. 2018; Scholtens et al. 2014; Gall et al. 2010; Anderson et al. 2014). In our study, “Central carbon metabolism in cancer”, “Protein digestion and absorption”, “Alanine, aspartate and glutamate metabolism”, “ABC transporters”, and “Aminoacyl-tRNA biosynthesis” pathways in HTR-8/SVneo cells were widely affected by CAMK4-overexpressing, which indicated that CAMK4 may activate metabolism of amino acid, lipid and carbohydrates in IR trophoblast cells. The normal amino acid metabolism is crucial for fetaplacental development (Regnault et al. 2005), and ABC transporters protect placental tissue from the cellular accumulation of cytotoxic compounds, to prevent inflammation or oxidative stress (Aye and Keelan 2013). The above evidences suggested that CAMK4-induced alterations of polar metabolites may contributes to normal placentation and pregnancy, which may provide novel insights for prevention and therapy of GDM. However, considering the complexity of metabolism in vivo, we could not determine whether the effects of CAMK4 overexpression on experimental animals and even humans are the same as those on cells. Koga et al*.* reported that CAMK4 inhibition decreased glycolysis in naïve CD^4+^ T cells from mice with systemic lupus erythematosus (Koga et al. 2019), and its enhanced expression promoted glucose uptake in muscle of mice (Lee et al. 2014). While suppressed glycolysis were found in placenta of pregnant women with hyperglycemia (Valent et al. 2021), suggesting that CAMK4 overexpression may be beneficial for glycometabolism regulation although its effectiveness and safety needed to be confirmed in experimental GDM animals.

## Conclusion

In summary, results from the present study reveal that CAMK4 overexpression faciliated proliferation and invasion in trophoblast cells under IR condition by activation of NUR77-mediated autophagy and insulin signaling. CAMK4 overexpression also induced metabolic alterations in IR HTR-8/SVneo cells. Although further exploration is still needed, our results indicate that the activation of CAMK4/NUR77 axis may contribute to the improvement of insulin sensitivity in trophoblast cells, which provides a potential target for GDM treatment.

## Supplementary Information

**Additional file 1: Figure S1.** HFD induced hyperglucemia and IR in mice during pregnancy.Maternal body weight was measured every six days.Fasting blood glucose and insulin levels were detectedat GD18.5 after fasting for 12 h. Homeostasis model assessment for insulin resistancewas used to estimate insulin resistance using the following formula: HOMA-IR = [fasting glucose × fasting insulin]/22.5. Error bars depicted the standard deviation of the mean. **p < 0.01, ***p < 0.001.

**Additional file 2: Figure S2. **IR inhibited EMT process in HTR-8/SVneo cells.The mRNA levels of epithelial markers, E-cadherin and CK7 in HTR-8/SVneo cells with or without IR induction.The mRNA levels of mesenchymal markers, vimentin and TGF-β1 in HTR-8/SVneo cells with or without IR induction. Error bars depicted the standard deviation of the mean. *p<0.05, **p < 0.01, ***p < 0.001.

**Additional file 3. **The primary DEMs analysis in positive ion mode.

**Additional file 4. **The primary DEMs analysis in negative ion mode.

**Additional file 5. **The identification of DEMs in HTR-8/SVneo cells.

## Data Availability

The datasets generated and/or analysed during the current study are not publicly available due [REASON WHY DATA ARE NOT PUBLIC] but are available from the corresponding author on reasonable request.
