# Supplementary figures and images for: Calcium/calmodulin dependent protein kinase IV in trophoblast cells under insulin resistance: functional and metabolomic analyses
Source: Mol Med. 2023 Jun 29;29:82. doi: 10.1186/s10020-023-00669-8 (PMC10308755; doi:10.1186/s10020-023-00669-8)

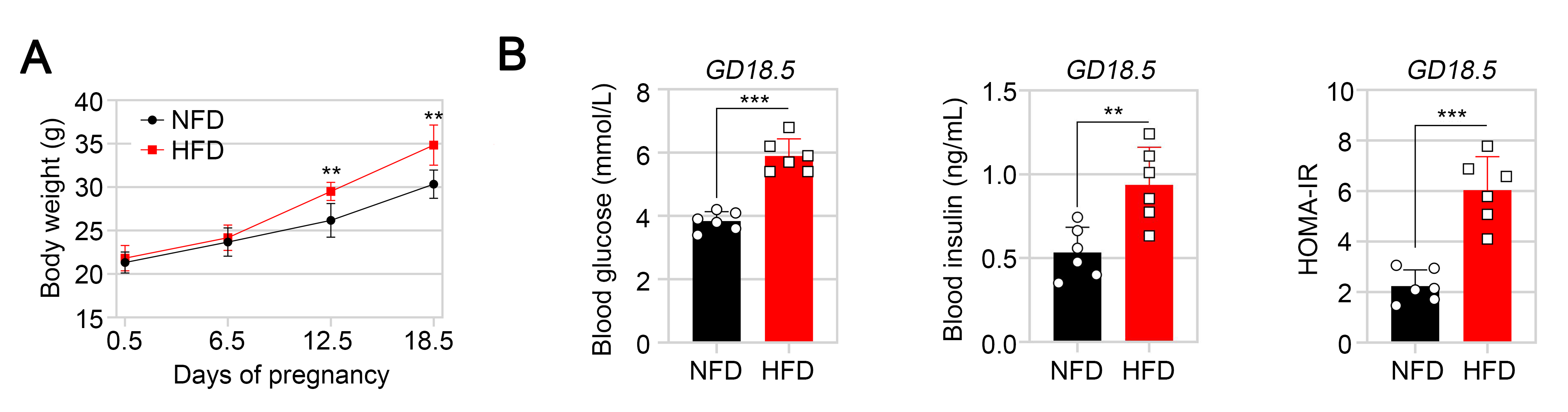

Supplement: Supplementary file 1 — Additional file 1: Figure S1. HFD induced hyperglucemia and IR in mice during pregnancy.Maternal body weight was measured every six days.Fasting blood glucose and insulin levels were detectedat GD18.5 after fasting for 12 h. Homeostasis model assessment for insulin resistancewas used to estimate insulin resistance using the following formula: HOMA-IR = [fasting glucose × fasting insulin]/22.5. Error bars depicted the standard deviation of the mean. **p < 0.01, ***p < 0.001. [file 10020_2023_669_MOESM1_ESM.tif]

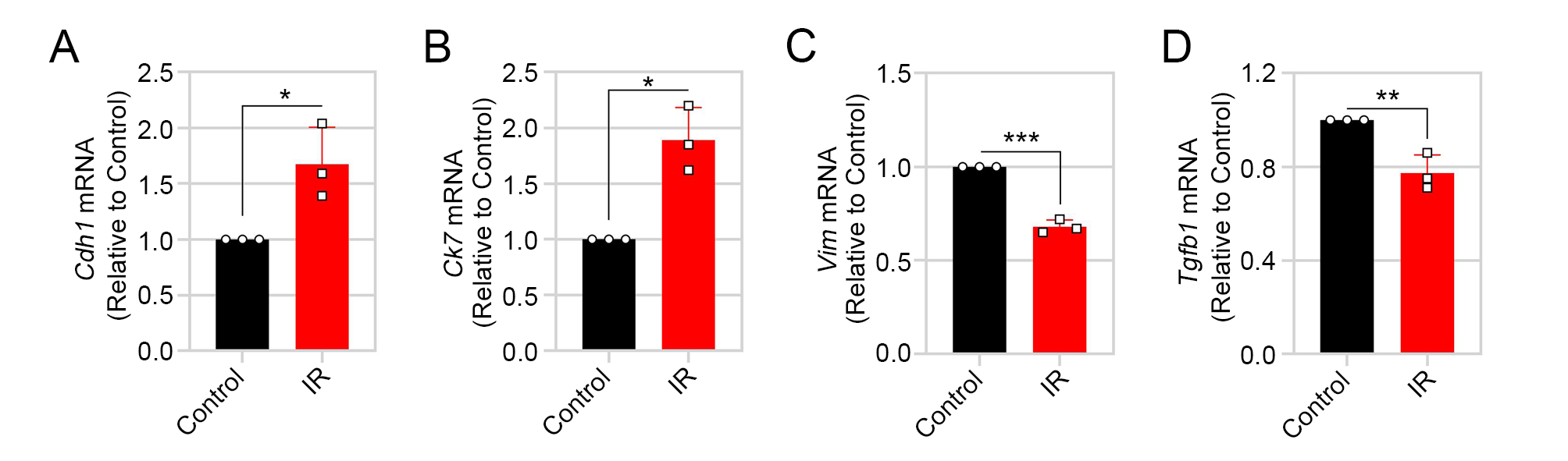

Supplement: Supplementary file 2 — Additional file 2: Figure S2. IR inhibited EMT process in HTR-8/SVneo cells.The mRNA levels of epithelial markers, E-cadherin and CK7 in HTR-8/SVneo cells with or without IR induction.The mRNA levels of mesenchymal markers, vimentin and TGF-β1 in HTR-8/SVneo cells with or without IR induction. Error bars depicted the standard deviation of the mean. *p<0.05, **p < 0.01, ***p < 0.001. [file 10020_2023_669_MOESM2_ESM.tif]
